# Supplementary material for: Unveiling the Bioactive Potential of the Invasive Jellyfish Phyllorhiza punctata Through Integrative Transcriptomic and Proteomic Analyses
Source: Biomolecules. 2025 Aug 4;15(8):1121. doi: 10.3390/biom15081121 (PMC12383608; doi:10.3390/biom15081121)
Supplement: Supplementary file 1 [file biomolecules-15-01121-s001.zip › Suplementary Material/FileS3/PEPFOLD4/XP_065067106_13975/clusters.html]

 PEP-FOLD clusters


Download archive of all models  here   

|  |  |  |
| --- | --- | --- |
| # | file | sOPEP |
| # cluster 1 - Size: 52 |  |  |
|  | XP\_065067106\_13975-model1 | -15.3696 |
|  | XP\_065067106\_13975-model1.1 | -15.3696 |
|  | XP\_065067106\_13975-model1.2 | -15.3696 |
|  | XP\_065067106\_13975-model1.3 | -15.2495 |
|  | XP\_065067106\_13975-model1.4 | -15.2495 |
|  | XP\_065067106\_13975-model1.5 | -15.2495 |
|  | XP\_065067106\_13975-model1.6 | -15.2495 |
|  | XP\_065067106\_13975-model1.7 | -15.2495 |
|  | XP\_065067106\_13975-model1.8 | -15.2495 |
|  | XP\_065067106\_13975-model1.9 | -15.2495 |
|  | XP\_065067106\_13975-model1.10 | -15.2495 |
|  | XP\_065067106\_13975-model1.11 | -15.2495 |
|  | XP\_065067106\_13975-model1.12 | -15.2495 |
|  | XP\_065067106\_13975-model1.13 | -15.2495 |
|  | XP\_065067106\_13975-model1.14 | -15.2115 |
|  | XP\_065067106\_13975-model1.15 | -15.2115 |
|  | XP\_065067106\_13975-model1.16 | -15.2115 |
|  | XP\_065067106\_13975-model1.17 | -15.2115 |
|  | XP\_065067106\_13975-model1.18 | -15.2115 |
|  | XP\_065067106\_13975-model1.19 | -15.2115 |
|  | XP\_065067106\_13975-model1.20 | -15.2115 |
|  | XP\_065067106\_13975-model1.21 | -15.2115 |
|  | XP\_065067106\_13975-model1.22 | -15.2115 |
|  | XP\_065067106\_13975-model1.23 | -15.2115 |
|  | XP\_065067106\_13975-model1.24 | -15.2115 |
|  | XP\_065067106\_13975-model1.25 | -15.2115 |
|  | XP\_065067106\_13975-model1.26 | -15.2115 |
|  | XP\_065067106\_13975-model1.27 | -15.2115 |
|  | XP\_065067106\_13975-model1.28 | -15.2115 |
|  | XP\_065067106\_13975-model1.29 | -15.2115 |
|  | XP\_065067106\_13975-model1.30 | -15.2115 |
|  | XP\_065067106\_13975-model1.31 | -15.196 |
|  | XP\_065067106\_13975-model1.32 | -15.196 |
|  | XP\_065067106\_13975-model1.33 | -15.196 |
|  | XP\_065067106\_13975-model1.34 | -15.196 |
|  | XP\_065067106\_13975-model1.35 | -15.196 |
|  | XP\_065067106\_13975-model1.36 | -15.196 |
|  | XP\_065067106\_13975-model1.37 | -15.196 |
|  | XP\_065067106\_13975-model1.38 | -15.196 |
|  | XP\_065067106\_13975-model1.39 | -15.196 |
|  | XP\_065067106\_13975-model1.40 | -15.196 |
|  | XP\_065067106\_13975-model1.41 | -15.196 |
|  | XP\_065067106\_13975-model1.42 | -15.196 |
|  | XP\_065067106\_13975-model1.43 | -15.196 |
|  | XP\_065067106\_13975-model1.44 | -15.196 |
|  | XP\_065067106\_13975-model1.45 | -15.196 |
|  | XP\_065067106\_13975-model1.46 | -15.196 |
|  | XP\_065067106\_13975-model1.47 | -15.1572 |
|  | XP\_065067106\_13975-model1.48 | -15.1507 |
|  | XP\_065067106\_13975-model1.49 | -15.1507 |
|  | XP\_065067106\_13975-model1.50 | -15.1507 |
|  | XP\_065067106\_13975-model1.51 | -15.1507 |
| # cluster 2 - Size: 16 |  |  |
|  | XP\_065067106\_13975-model2 | -15.2678 |
|  | XP\_065067106\_13975-model2.1 | -15.2678 |
|  | XP\_065067106\_13975-model2.2 | -15.2678 |
|  | XP\_065067106\_13975-model2.3 | -15.2678 |
|  | XP\_065067106\_13975-model2.4 | -15.2678 |
|  | XP\_065067106\_13975-model2.5 | -15.2059 |
|  | XP\_065067106\_13975-model2.6 | -15.2059 |
|  | XP\_065067106\_13975-model2.7 | -15.2059 |
|  | XP\_065067106\_13975-model2.8 | -15.2059 |
|  | XP\_065067106\_13975-model2.9 | -15.2059 |
|  | XP\_065067106\_13975-model2.10 | -15.2059 |
|  | XP\_065067106\_13975-model2.11 | -15.2059 |
|  | XP\_065067106\_13975-model2.12 | -15.1379 |
|  | XP\_065067106\_13975-model2.13 | -15.1379 |
|  | XP\_065067106\_13975-model2.14 | -15.1379 |
|  | XP\_065067106\_13975-model2.15 | -15.0734 |
| # cluster 3 - Size: 15 |  |  |
|  | XP\_065067106\_13975-model3 | -15.2586 |
|  | XP\_065067106\_13975-model3.1 | -15.2586 |
|  | XP\_065067106\_13975-model3.2 | -15.2433 |
|  | XP\_065067106\_13975-model3.3 | -15.2433 |
|  | XP\_065067106\_13975-model3.4 | -15.2433 |
|  | XP\_065067106\_13975-model3.5 | -15.2433 |
|  | XP\_065067106\_13975-model3.6 | -15.2433 |
|  | XP\_065067106\_13975-model3.7 | -15.2433 |
|  | XP\_065067106\_13975-model3.8 | -15.2433 |
|  | XP\_065067106\_13975-model3.9 | -15.2433 |
|  | XP\_065067106\_13975-model3.10 | -15.2433 |
|  | XP\_065067106\_13975-model3.11 | -15.1642 |
|  | XP\_065067106\_13975-model3.12 | -15.1642 |
|  | XP\_065067106\_13975-model3.13 | -15.1434 |
|  | XP\_065067106\_13975-model3.14 | -15.1434 |
| # cluster 4 - Size: 9 |  |  |
|  | XP\_065067106\_13975-model4 | -15.2441 |
|  | XP\_065067106\_13975-model4.1 | -15.2441 |
|  | XP\_065067106\_13975-model4.2 | -15.2441 |
|  | XP\_065067106\_13975-model4.3 | -15.2441 |
|  | XP\_065067106\_13975-model4.4 | -15.1471 |
|  | XP\_065067106\_13975-model4.5 | -15.1471 |
|  | XP\_065067106\_13975-model4.6 | -15.1471 |
|  | XP\_065067106\_13975-model4.7 | -15.1471 |
|  | XP\_065067106\_13975-model4.8 | -15.1424 |
| # cluster 5 - Size: 2 |  |  |
|  | XP\_065067106\_13975-model5 | -15.1695 |
|  | XP\_065067106\_13975-model5.1 | -15.1695 |
| # cluster 6 - Size: 6 |  |  |
|  | XP\_065067106\_13975-model6 | -15.149 |
|  | XP\_065067106\_13975-model6.1 | -15.149 |
|  | XP\_065067106\_13975-model6.2 | -15.1485 |
|  | XP\_065067106\_13975-model6.3 | -15.1485 |
|  | XP\_065067106\_13975-model6.4 | -15.1485 |
|  | XP\_065067106\_13975-model6.5 | -15.1485 |
|  |  |  |
